# Is chlamydia screening and testing in Britain reaching young adults at risk of infection? Findings from the third National Survey of Sexual Attitudes and Lifestyles (Natsal-3)

**DOI:** 10.1136/sextrans-2015-052013

**Published:** 2015-08-19

**Authors:** Sarah C Woodhall, Kate Soldan, Pam Sonnenberg, Catherine H Mercer, Soazig Clifton, Pamela Saunders, Filomeno da Silva, Sarah Alexander, Kaye Wellings, Clare Tanton, Nigel Field, Andrew J Copas, Catherine A Ison, Anne M Johnson

**Affiliations:** 1 Research Department of Infection and Population Health, University College London, London, UK; 2 Centre for Infectious Disease Surveillance & Control (CIDSC), Public Health England, London, UK; 3 NatCen Social Research, London, UK; 4 Microbiology Services, Public Health England, London, UK; 5 Department of Social and Environmental Health Research, London School of Hygiene and Tropical Medicine, London, UK

**Keywords:** CHLAMYDIA TRACHOMATIS, SCREENING, EPIDEMIOLOGY (GENERAL)

## Abstract

**Background:**

In the context of widespread opportunistic chlamydia screening among young adults, we aimed to quantify chlamydia testing and diagnosis among 16–24 year olds in Britain in relation to risk factors for prevalent chlamydia infection.

**Methods:**

Using data from sexually experienced (≥1 lifetime sexual partner) 16-year-old to 24-year-old participants in Britain's third National Survey of Sexual Attitudes and Lifestyles (conducted 2010–2012), we explored socio-demographic and behavioural factors associated with prevalent chlamydia infection (detected in urine; n=1832), self-reported testing and self-reported diagnosis in the last year (both n=3115).

**Results:**

Chlamydia prevalence was 3.1% (95% CI 2.2% to 4.3%) in women and 2.3% (1.5% to 3.4%) in men. A total of 12.3% of women and 5.3% men had a previous chlamydia diagnosis. Factors associated with prevalent infection were also associated with testing and diagnosis (eg, increasing numbers of sexual partners), with some exceptions. For example, chlamydia prevalence was higher in women living in more deprived areas, whereas testing was not. In men, prevalence was higher in 20–24 than 16–19 year olds but testing was lower. Thirty per cent of women and 53.7% of men with ≥2 new sexual partners in the last year had not recently tested.

**Conclusions:**

In 2010–2012 in Britain, the proportion of young adults reporting chlamydia testing was generally higher in those reporting factors associated with chlamydia. However, many of those with risk factors had not been recently tested, leaving potential for undiagnosed infections. Greater screening and prevention efforts among individuals in deprived areas and those reporting risk factors for chlamydia may reduce undiagnosed prevalence and transmission.

## Introduction

*Chlamydia trachomatis* (‘chlamydia’) is the most commonly diagnosed sexually transmitted infection (STI) in the UK.^1^ Most chlamydia infections are asymptomatic, and untreated infections can cause serious complications including pelvic inflammatory disease, ectopic pregnancy and tubal factor infertility in women.^2^ By diagnosing and treating asymptomatic infections, chlamydia screening potentially reduces the risk of complications^3^ and is expected to reduce chlamydia prevalence and transmission.^4^ In England, the National Chlamydia Screening Programme (NCSP) recommends that sexually active under 25 year olds are tested annually and on change of sexual partner.^5^ Chlamydia screening is offered opportunistically in clinical and non-clinical settings in England. Scotland and Wales do not have an organised screening programme; guidelines recommend asymptomatic testing of young adults^6–8^ with a focus on those at high risk (eg, those reporting multiple sexual partners in the last year, those with a previous diagnosis or patients attending genitourinary medicine (GUM) clinics).

Chlamydia testing of young adults increased substantially in the UK over the last decade. Increases in testing occurred in GUM clinics as a result of improved access to sexual health services^9–11^ and availability of diagnostic testing using non-invasive samples.^12^ In England, a major increase was driven by the national scale-up of the NCSP. After a phased roll-out from 2003 to 2008, a step change in screening activity outside of GUM clinics was seen from 2008 to 2010 as local areas responded to national targets for testing coverage.^13^ Testing coverage (number of tests divided by total 15-year-old to 24-year-old population) peaked at 34% in 2010 and fell slightly to 30% and 26% in 2011 and 2012, respectively.^14^ ^15^

The third National Survey of Sexual Attitudes and Lifestyles (Natsal-3) is a stratified cross-sectional probability sample survey of adults resident in Britain (England, Scotland and Wales; Northern Ireland was not included).^16^ Conducted from 2010 to 2012, Natsal-3 included anonymous testing of urine specimens for STI, including chlamydia, and asked questions on chlamydia testing and diagnosis history. The survey provides a unique opportunity to investigate patterns of chlamydia infection and testing within a nationally representative sample of the British population.

Sonnenberg *et al* previously reported an overview of STI prevalence and service use using data from Natsal-3.^17^ Chlamydia prevalence in 16–44 year olds was 1.5% in women and 1.1% in men and was higher among 16–24 year olds (women: 3.1%; men: 2.3%). Among 16–24 year olds, 54.2% of women and 34.6% of men reported testing in the last year. Although prevalence was reported by age group, factors associated with prevalent infection were assessed among all 16–44 year olds. Only a limited number of factors associated with chlamydia prevalence and testing were explored (age group, area-level deprivation, sexual partners in the last year, sexual partners in the last year without a condom (investigated for prevalence only), age at first sex and any same-sex experience). In this paper, we report a detailed analysis among 16–24 year olds in Britain as this is the age group targeted by the NCSP in England. We describe and compare factors associated with prevalent chlamydia infection, previous chlamydia diagnosis and chlamydia testing to assess the extent to which opportunistic chlamydia screening is reaching young adults at risk of chlamydia.

## Methods

### Participants and procedures

In Natsal-3, participants were interviewed using computer-assisted face-to-face and computer-assisted self-interview for the most sensitive questions. The overall response rate was 57.7%, in line with other major social surveys conducted in Britain around the same time,^18^ ^19^ achieving a sample of 15 162 16–74 year olds.^16^ A subset of participants, including all 16–17 year olds (regardless of reported sexual activity) and 18–24 year olds who reported at least one sexual partner by the time of the interview (hereafter termed ‘sexually experienced’) were invited to provide a urine sample for anonymous STI testing.^16^ ^17^ Participants did not receive their test results.^20^ Of all Natsal-3 respondents eligible for the urine study, 57% provided a sample. Urine samples were posted to Public Health England where they were batch-tested for chlamydia using the Aptima Combo 2 assay (Hologic Gen-Probe); positive and equivocal results were confirmed with the Aptima chlamydia monospecific assay.^17^ Details of the survey methods and questionnaire are available elsewhere.^16^

We estimated the prevalence of chlamydia detected in urine (hereafter termed ‘prevalent infection’), self-reported chlamydia test in the last year (‘recent testing’), self-reported chlamydia diagnosis in the last year (‘recent diagnosis’) and self-reported chlamydia diagnosis ever.

A flow chart of participants included in our analyses is presented in the online supplementary material. Analyses of recent testing and recent diagnosis were based on sexually experienced 16–24 year olds (n=3115). Analyses of prevalent infection were among those who provided a urine sample for STI testing and for whom a valid chlamydia test result is available (n=1832, 62 of whom had a prevalent infection).

### Statistical analyses

Analyses were carried out using Stata V.12.1, accounting for weighting, clustering and stratification of the data. Survey weights were applied to adjust for unequal probability of selection and non-response to make the sample data broadly representative of the British general population, according to the 2011 Census, in terms of sex, age group and Government Office Region.^16^ Willingness to provide a urine sample varied by demographic and behavioural variables, including age, number of sexual partners (by the time of the interview/without a condom in the last year), same-sex experience and sexual health clinic attendance. Estimates of prevalent infection were therefore given an additional weight to reduce bias in the profile of urine sample respondents.^16^ ^21^

Factors associated with prevalent infection, recent diagnosis and recent testing were investigated using univariable and multivariable logistic regression, for women and men separately. Although the overall percentage diagnosed with chlamydia (ever or in the last year) was estimated among the sexually experienced population, risk factors for recent diagnosis were investigated among those with a recent test to investigate associations with being infected at the time of testing rather than with testing per se. Socio-demographic and behavioural factors previously demonstrated to be associated with STI risk were included as predictor variables.^22–24^ Associations with deprivation were explored using both residence-based (quintile of Index of Multiple Deprivation (IMD) for the lower layer super output area (LSOA) of residence (a geographical area of around 1500 people^25^)) and individual-based (age left school) measures. Sexual behaviours investigated included numbers of sexual partners in the last year (total, new, without a condom), number of sexual partners by the time of the interview (hereafter ‘lifetime sexual partners’) and condom use at last sex. Frequency of binge drinking was included as a proxy for sexual risk behaviour that may not be captured in reported numbers of sexual partners.

With two exceptions, all variables included in univariable models were included in multivariable models: number of sexual partners in the last year was not included due to collinearity with other sexual partnership variables; age left school was not included as data were unavailable for 16 year olds.

To explore how chlamydia infections were distributed across population subgroups, we calculated the percentage reporting selected socio-demographic and behavioural factors among (a) individuals with a prevalent infection, (b) individuals recently diagnosed and (c) the sexually experienced population.

## Results

Table 1 shows chlamydia prevalence and self-reported chlamydia testing and diagnosis in the last year among sexually experienced 16–24 year olds. Around two-thirds (62.5%) of women and 43.2% of men had either been tested or offered a test in the last year. A total of 12.3% of women and 5.3% men had ever been diagnosed with chlamydia.

**Table 1:** Prevalence of chlamydia infection detected in urine and of self-reported testing and diagnosis by sex (sexually experienced 16–24 year olds)

	Women		Men		Denominator* (weighted, unweighted)	
	%	95% CI	%	95% CI	Women	Men
Prevalent chlamydia infection detected in urine	3.1	2.2 to 4.3	2.3	1.5 to 3.4	597, 992	625, 840
Tested for chlamydia in the last year	54.2	51.4 to 56.9	34.6	31.9 to 37.4	966, 1736	1003, 1375
Offered, not tested for chlamydia in the last year	8.3	6.9 to 9.9	8.6	7.0 to 10.4	966, 1735	1001, 1373
Diagnosed with chlamydia in the last year	3.0	2.2 to 4.0	2.0	1.3 to 3.0	962, 1727	992, 1364
Ever diagnosed with chlamydia	12.3	10.6 to 14.1	5.3	4.1 to 6.7	962, 1727	992, 1364

*Denominators for recent testing/offer of testing and for diagnosis (recent or ever) differ due to item-missingness.

95% CI of unadjusted OR and p values for unadjusted and adjusted OR are presented in full in the online supplementary material.

Among those recently tested, <10% reported a clinical indication (symptoms; a partner with chlamydia/symptoms; check-up after a previous diagnosis) for their last test. Around three-quarters of women and half of men had last been tested in a sexual health clinic, general practice (GP) surgery or family planning clinic. Almost all (95.4%) individuals recently diagnosed had most recently been tested in one of these settings. Half of those recently diagnosed had last been tested due to symptoms or having a partner with chlamydia/symptoms (table 2).

**Table 2:** Reason and location of most recent chlamydia test, among those tested for chlamydia in the last year, by sex and by whether diagnosed in last year (sexually experienced 16–24 year olds)

	By sex				By whether diagnosed in the last year*			
	Women		Men		Diagnosed in the last year		Not diagnosed in the last year	
	%	95% CI	%	95% CI	%	95% CI	%	95% CI
Denominator (weighted, unweighted)	523, 943		347, 475		48, 81		816, 1330	
Reason for most recent test								
Had symptoms	4.2	3.0 to 5.8	4.2	2.7 to 6.5	29.0	19.0 to 41.5	2.7	1.9 to 3.8
Partner diagnosed with chlamydia or had symptoms	2.8	1.7 to 4.5	3.8	2.4 to 6.1	20.9	12.8 to 32.2	2.2	1.4 to 3.4
Check up after a previous positive	1.3	0.63 to 2.6	0.95	0.33 to 2.7	8.6	3.2 to 21.1	0.7	0.4 to 1.4
Wanted a check-up/offered a test/worried about risk	84.9	82.1 to 87.4	87.3	83.8 to 90.1	37.2	26.2 to 49.86	88.7	86.8 to 90.4
Other	6.8	5.3 to 8.7	3.7	2.3 to 6.0	4.3	1.5 to 12.0	5.7	4.5 to 7.1
Location of most recent chlamydia test								
Sexual health clinic	28.9	25.5 to 32.6	30.5	25.9 to 35.5	62.9	50.4 to 73.9	27.6	25.0 to 30.4
GP surgery	35.1	31.7 to 38.6	17.0	13.6 to 20.9	27.1	17.7 to 39.1	28.0	25.3 to 30.8
NHS Family Planning clinic	9.2	7.4 to 11.4	4.3	2.7 to 6.8	5.4	1.6 to 16.3	7.3	6.0 to 8.9
School, college or university	11.6	9.4 to 14.2	24.5	20.4 to 29.1	1.7	0.4 to 7.2	17.5	15.2 to 20.1
Elsewhere	15.2	12.9 to 17.8	23.8	19.3 to 28.9	2.9	1.0 to 8.1	19.6	17.2 to 22.2

*Women and men were combined due to small denominator for diagnosed in the last year.

95% CI of unadjusted OR and p values for unadjusted and adjusted OR are presented in full in the online supplementary material.

GP, general practice; NHS, National Health Service.

Tables 3 and 4 explore the associations between socio-demographic and behavioural variables and prevalent infection, recent testing and recent diagnosis. In univariable analyses, higher numbers of sexual partners (total/new/without a condom) in the last year were significantly (p<0.05) associated with prevalent infection among women and men. In women, area-level deprivation (measured at LSOA level) and frequency of binge drinking were also associated with prevalent infection. Among men, number of lifetime sexual partners, age group, age left school, age at first sex and condom non-use at last sex were significantly associated with prevalent infection. Similar factors were associated with recent diagnosis among those tested. In multivariable analyses, living in more deprived areas and more frequent binge drinking remained significantly associated with having a prevalent infection in women. Older age group, living in more deprived areas and higher numbers of lifetime sexual partners remained significantly associated with prevalent infection in men.

**Table 3:** Percentage, unadjusted and adjusted ORs for prevalent chlamydia infection, self-reported diagnosis in the last year and self-reported testing by socio-demographic and behavioural factors (sexually experienced 16–24 year old women)

	Prevalent infection detected in urine (n=992)					Diagnosed with chlamydia in the last year (among those tested in the last year) (n=940)					Tested for chlamydia in the last year (n=1736)					Denominator (weighted, unweighted)*		
	%	95% CI	OR	AOR†	95% CI	%	95% CI	OR	AOR†	95% CI	%	95% CI	OR	AOR†	95% CI	Infection	Diagnosis	Tested
Age group																		
16–19	3.8	2.2 to 6.3	1.00	1.00	–	6.0	3.8 to 9.2	1.00	1.00	–	56.6	52.5 to 60.6	1.00	1.00	–	214, 395	193, 375	343, 672
20–24	2.7	1.7 to 4.3	0.71	0.71	0.27 to 1.87	5.1	3.4 to 7.6	0.86	0.80	0.35 to 1.78	52.8	49.2 to 56.4	0.86	0.82	0.62 to 1.07	383, 597	329, 565	623, 1064
Country‡																		
England	2.9	2.0 to 4.3	1.00	1.00	–						57.1	54.1 to 60.1	**1.00**	**1.00**	–	504, 817	469, 832	823, 1452
Scotland	3.1	1.1 to 8.6	1.08	1.34	0.43 to 4.14						32.4	24.4 to 41.5	**0.36**	**0.29**	**0.18 to 0.45**	56, 103	30, 58	91, 178
Wales	5.3	1.9 to 13.8	1.87	1.88	0.63 to 5.54						45.6	36.2 to 55.4	**0.63**	**0.53**	**0.32 to 0.85**	37, 72	24, 50	52, 106
IMD quintile of LSOA of residence§																		
2 least deprived	1.3	0.5 to 3.4	**1.00**	**1.00**	–	4.8	2.8 to 8.1	1.00	1.00	–	54.2	49.5 to 58.8	1.00	1.00	–	213, 355	183, 319	338, 595
Middle quintile	1.8	0.8 to 4.2	**1.37**	**1.40**	**0.39 to 4.98**	3.5	1.6 to 7.3	0.71	1.06	0.37 to 3.04	54.4	48.0 to 60.7	1.01	1.03	0.71 to 1.48	111, 174	102, 176	189, 324
2 most deprived	4.9	3.3 to 7.3	**3.82**	**4.23**	**1.53 to 11.6**	6.8	4.6 to 10.0	1.46	1.70	0.73 to 3.91	54.0	49.8 to 58.2	0.99	0.97	0.73 to 1.29	273, 463	236, 445	439, 817
Age left school¶††																		
17+	3.2	2.1 to 4.8	1.00			5.2	3.6 to 7.5	1.00			54.3	51.0 to 57.6	1.00			445, 700	387, 658	715, 1217
16	3.4	1.9 to 6.0	1.06			6.2	3.7 to 10.5	1.21			55.5	50.1 to 60.8	1.05			120, 229	109, 228	196, 405
Age at first heterosexual sex																		
17+	1.6	0.7 to 3.7	1.00	1.00	–	4.1	1.8 to 8.9	1.00	1.00	–	43.9	38.7 to 49.1	**1.00**	1.00	–	188, 246	137, 215	313, 489
16	3.9	2.1 to 6.9	2.52	2.20	0.67 to 7.17	5.0	2.8 to 8.9	1.24	0.80	0.26 to 2.42	56.4	51.6 to 61.1	**1.66**	1.39	0.99 to 1.92	178, 304	154, 272	273, 503
<16	4.0	2.4 to 6.6	2.65	1.82	0.60 to 5.42	6.9	4.8 to 9.9	1.76	0.89	0.35 to 2.25	63.9	59.8 to 67.9	**2.27**	1.44	1.05 to 1.97	213, 415	220, 429	344, 680
Number of sexual partners in the last year††																		
0 or 1	2.5	1.5 to 4.0	**1.00**			3.0	1.7 to 5.4	**1.00**			46.6	43.3 to 50.0	**1.00**			387, 600	291, 507	624, 1096
2	3.9	1.8 to 8.5	**1.62**			6.6	3.7 to 11.6	**2.25**			65.2	58.8 to 71.1	**2.15**			90, 161	93, 178	143, 275
3–4	1.9	0.7 to 5.1	**0.75**			8.8	5.2 to 14.6	**3.08**			69.7	62.0 to 76.4	**2.63**			63, 127	76, 146	111, 210
5+	8.3	3.9 to 16.8	**3.57**			11.6	6.2 to 20.8	**4.19**			74.8	64.4 to 83.0	**3.40**			49, 93	58, 101	77, 135
Number of new sexual partners in the last year																		
0	2.2	1.3 to 3.7	**1.00**	1.00	–	2.8	1.5 to 5.0	**1.00**	1.00	–	45.6	41.9 to 49.3	**1.00**	**1.00**	–	313, 495	226, 397	495, 873
1	2.8	1.2 to 6.3	**1.26**	1.17	0.38 to 3.52	4.8	2.5 to 9.1	**1.76**	1.89	0.69 to 5.16	59.2	54.0 to 64.2	**1.73**	**1.69**	**1.25 to 2.27**	160, 263	156, 287	264, 485
2+	5.9	3.5 to 9.8	**2.73**	1.65	0.50 to 5.39	10.7	7.3 to 15.6	**4.23**	3.09	1.07 to 8.86	70.0	63.8 to 75.6	**2.79**	**1.46**	**0.95 to 2.21**	118, 225	137, 249	197, 359
Number of sexual partners in the last year without a condom																		
0	2.9	1.2 to 7.1	**1.00**	1.00	–	4.2	1.5 to 11.6	**1.00**	1.00	–	36.4	30.8 to 42.3	**1.00**			120, 173	76, 130	210, 361
1	2.2	1.4 to 3.6	**0.76**	0.34	0.10 to 1.10	3.8	2.4 to 6.1	**0.90**	0.72	0.15 to 3.21	54.7	51.4 to 58.0	**2.11**	**1.52**	**1.03 to 2.24**	368, 606	319, 567	585, 1049
2+	6.3	3.5 to 11.2	**2.25**	0.49	0.12 to 1.83	10.3	7.0 to 15.0	**2.60**	0.90	0.18 to 4.49	74.2	68.3 to 79.4	**5.04**	**1.86**	**1.09 to 3.15**	108, 212	126, 241	169, 322
Number of lifetime sexual partners																		
1–4	2.4	1.4 to 4.2	**1.00**	1.00	–	2.1	1.1 to 4.0	**1.00**	1.00	–	43.5	40.0 to 47.1	**1.00**	1.00	–	321, 482	218, 391	503, 894
5–9	2.8	1.4 to 5.2	**1.15**	0.87	0.32 to 2.32	6.2	3.7 to 10.3	**3.07**	2.40	0.90 to 6.37	63.8	58.5 to 68.7	**2.29**	1.96	1.43 to 2.69	150, 267	161, 289	252, 453
10+	5.4	2.9 to 9.7	**2.29**	1.39	0.45 to 4.18	9.9	6.5 to 14.7	**5.12**	3.76	1.19 to 11.8	69.6	63.9 to 74.7	**2.97**	2.11	1.41 to 3.13	121, 234	141, 254	202, 373
Condom used for most recent sex with most recent partner																		
Yes	2.8	1.5 to 5.3	1.00	1.00	–	3.4	1.6 to 7.0	1.00	1.00	–	51.6	46.9 to 56.3	**1.00**	1.00	–	202, 314	170, 303	330, 586
No	3.6	2.4 to 5.4	1.28	1.59	0.67 to 3.74	6.3	4.4 to 8.9	1.90	1.88	0.81 to 4.32	58.0	54.6 to 61.3	**1.30**	1.01	0.74 to 1.35	352, 613	328, 592	567, 1027
Concurrent partnerships in last year**																		
No	2.7	1.8 to 4.2	1.00	1.00	–	4.3	2.9 to 6.4	1.00	1.00	–	51.0	47.8 to 54.2	**1.00**	1.00	–	439, 706	361, 639	710, 1256
Yes	6.3	2.9 to 13.4	2.40	1.34	0.48 to 3.70	8.1	4.1 to 15.3	1.95	0.75	0.27 to 2.00	74.6	67.1 to 80.8	**2.81**	1.46	0.93 to 2.28	66, 134	78, 146	105, 196
Unknown	3.1	1.3 to 7.2	1.14	1.13	0.38 to 3.31	7.8	4.3 to 13.9	1.88	1.46	0.62 to 3.38	64.6	57.5 to 71.2	**1.76**	1.53	1.02 to 2.28	73, 127	76, 144	117, 226
Frequency of binge drinking																		
Never/≤monthly	2.5	1.5 to 4.1	**1.00**	**1.00**	–	3.7	2.4 to 5.7	**1.00**	1.00	–	52.1	48.7 to 55.5	**1.00**	1.00	–	373, 598	312, 561	601, 1085
Monthly	1.2	0.5 to 3.3	**0.49**	**0.46**	**0.13 to 1.51**	6.6	3.6 to 11.8	**1.85**	1.91	0.74 to 4.89	52.4	46.6 to 58.2	**1.01**	0.75	0.54 to 1.02	130, 226	112, 200	214, 375
≥Weekly	7.9	4.7 to 13.1	**3.35**	**2.51**	**1.08 to 5.76**	9.4	5.2 to 16.3	**2.69**	2.06	0.75 to 5.61	64.4	57.6 to 70.7	**1.66**	1.16	0.81 to 1.64	95, 168	97, 177	151, 274
Ever had any same sex experience/ contact																		
No	3.1	2.1 to 4.4	1.00	1.00	–	5.3	3.7 to 7.5	1.00	1.00	–	51.9	48.8 to 55.0	**1.00**	1.00	–	473, 750	388, 704	749, 1352
Yes	3.2	1.4 to 7.1	1.05	0.74	0.30 to 1.76	5.9	3.5 to 10.0	1.13	0.71	0.30 to 1.68	61.9	56.0 to 67.5	**1.51**	1.21	0.88 to 1.65	124, 242	134, 236	218, 384

Variables in bold indicate those which are statistically significant (p<0.05).

95% CI of unadjusted OR and p values for unadjusted and adjusted OR are presented in full in the online supplementary material.

*N in column headings shows unweighted denominators. Total denominators by characteristic and in multivariable models vary due to item-missingness.

†AOR adjusted for all variables shown.

‡Results for recent diagnosis are not reported due to small sample size in Scotland and Wales when limited to those tested.

§IMD of LSOA of residence. IMD scores for England, Scotland and Wales were adjusted before being combined and assigned to quintiles, using the method described by Payne and Abel.^25^

¶Excludes 16 year olds.

**Among those with ≥1 sexual partner in last year.

††With two exceptions, all variables included in univariable models were included in multivariable models: number of sexual partners in the last year was not included due to collinearity with other sexual partnership variables; age left school was not included as data were unavailable for 16 year-olds.

AOR, adjusted OR; IMD, Index of Multiple Deprivation; LSOA, lower super output area.

**Table 4:** Percentage, unadjusted and adjusted ORs for prevalent chlamydia infection, self-reported diagnosis in the last year and self-reported testing by socio-demographic and behavioural factors (sexually experienced 16–24 year old men)

	Prevalent infection detected in urine (n=840)					Diagnosed with chlamydia in the last year (among those tested in the last year) (n=471)					Tested for chlamydia in the last year (n=1375)					Denominator (weighted, unweighted)*		
	%	95% CI	OR	AOR†	95% CI	%	95% CI	OR	AOR†	95% CI	%	95% CI	OR	AOR†	95% CI	Infection	Diagnosis	Tested
Age group																		
16–19	0.3%	0.1 to 1.4	**1.00**	**1.00**	–	4.7%	2.4 to 9.0	1.00	1.00	–	40.4%	35.9 to 45.1	**1.00**	**1.00**	–	234, 343	151, 226	374, 582
20–24	3.4%	2.2 to 5.2	**10.6**	**7.54**	**1.37 to 41.3**	6.7%	3.9 to 11.1	1.46	0.76	0.26 to 2.15	31.1%	27.8 to 34.7	**0.67**	**0.53**	**0.37 to 0.73**	391, 497	192, 245	629, 793
Country‡																		
England	1.9%	1.2 to 3.0	1.00	1.00	–						37.3%	34.3 to 40.3	**1.00**	**1.00**	–	532, 719	316, 440	859, 1181
Scotland	5.7%	2.1 to 14.3	3.13	3.16	0.78 to 12.8						22.2%	14.0 to 33.5	**0.48**	**0.33**	**0.16 to 0.64**	60, 72	20, 22	89, 111
Wales	1.7%	0.2 to 12.1	0.88	1.20	0.18 to 7.63						12.8%	6.9 to 22.3	**0.25**	**0.19**	**0.08 to 0.40**	33, 49	7, 9	55, 83
IMD quintile of LSOA of residence§																		
2 least deprived	1.3%	0.4 to 3.6	1.00	**1.00**	–	5.2%	2.5 to 10.5	1.00	1.00	–	34.5%	30.0 to 39.2	1.00	1.00	–	241, 315	127, 180	369, 509
Middle quintile	1.6%	0.6 to 4.4	1.24	**1.01**	**0.15 to 6.68**	5.0%	1.7 to 13.8	0.96	0.68	0.15 to 2.97	33.3%	27.4 to 39.9	0.95	1.04	0.70 to 1.52	114, 164	60, 86	183, 263
2 most deprived	3.4%	2.1 to 5.6	2.71	**3.75**	**1.11 to 12.5**	6.5%	3.6 to 11.4	1.26	1.06	0.42 to 2.64	35.2%	31.1 to 39.5	1.03	1.13	0.82 to 1.53	269, 361	155, 205	450, 603
Age left school¶††																		
17+	1.6%	0.9 to 2.7	**1.00**			5.3%	3.1 to 9.0	1.00			33.6%	30.4 to 37.1	1.00			439, 568	233, 304	703, 927
16	5.0%	2.7 to 9.2	**3.28**			7.2%	3.6 to 13.8	1.38			37.8%	32.3 to 43.5	1.20			143, 206	87, 134	230, 334
Age at first heterosexual sex																		
17+	1.0%	0.3 to 2.8	**1.00**	1.00	–	2.8%	0.7 to 9.9	1.00	1.00	–	25.6%	21.4 to 30.3	**1.00**	1.00	–	210, 245	87, 112	340, 431
16	1.5%	0.5 to 4.6	**1.49**	1.14	0.27 to 4.74	4.7%	1.8 to 11.8	1.75	1.14	0.24 to 5.30	33.4%	27.9 to 39.4	**1.46**	1.13	0.75 to 1.67	148, 205	84, 108	253, 351
<16	4.0%	2.4 to 6.5	**4.18**	1.65	0.55 to 4.90	7.8%	4.7 to 12.6	2.99	1.58	0.37 to 6.62	45.3%	40.7 to 49.9	**2.40**	1.53	1.07 to 2.19	238, 352	167, 243	376, 539
Number of sexual partners in the last year††																		
0 or 1	1.5%	0.7 to 3.0	**1.00**			3.4%	1.5 to 7.8	**1.00**			26.0%	22.6 to 29.7	**1.00**			359, 466	145, 196	568, 768
2	1.3%	0.4 to 4.2	**0.86**			0.9%	0.2 to 3.9	**0.27**			40.3%	33.2 to 47.7	**1.92**			123, 159	74, 99	185, 251
3–4	3.1%	1.1 to 8.5	**2.16**			1.5%	0.4 to 6.2	**0.44**			43.0%	35.5 to 50.9	**2.15**			70, 110	57, 83	134, 194
5+	7.5%	3.7 to 14.6	**5.47**			21.2%	12.9 to 32.7	**7.54**			60.9%	51.8 to 69.3	**4.42**			67, 100	63, 89	103, 146
Number of new sexual partners in the last year																		
0	1.8%	0.9 to 3.8	**1.00**	1.00	–	5.5%	2.3 to 12.6	1.00	1.00	–	26.0%	22.0 to 30.5	**1.00**	1.00	–	263, 335	108, 136	416, 540
1	0.8%	0.2 to 2.5	**0.42**	0.33	0.05 to 2.06	4.0%	1.7 to 9.0	0.71	1.13	0.09 to 13.9	36.7%	31.8 to 41.8	**1.64**	1.28	0.88 to 1.85	203, 270	115, 161	323, 452
2+	5.1%	2.9 to 8.8	**2.87**	0.47	0.09 to 2.45	8.0%	4.5 to 13.7	1.48	2.87	0.26 to 30.7	46.3%	40.7 to 52.0	**2.45**	1.06	0.67 to 1.68	152, 229	115, 170	251, 366
Number of sexual partners in the last year without a condom																		
0	0.3%	0.1 to 1.3	**1.00**	1.00	–	1.8%	0.4 to 8.4	**1.00**	1.00	–	27.0%	22.4 to 32.1	**1.00**	1.00	–	205, 248	88, 115	331, 450
1	1.7%	0.8 to 3.6	**5.26**	1.23	0.09 to 15.2	4.9%	2.6 to 8.9	**2.75**	0.78	0.19 to 3.15	34.3%	30.2 to 38.7	**1.41**	1.12	0.72 to 1.71	287, 396	160, 222	475, 640
2+	6.5%	3.9 to 10.9	**21.3**	4.95	0.42 to 57.9	10.9%	6.0 to 19.0	**6.51**	0.46	0.11 to 1.83	48.8%	42.3 to 55.4	**2.59**	1.37	0.80 to 2.34	130, 194	95, 134	194, 281
Number of lifetime sexual partners																		
1–4	0.4%	0.1 to 1.5	**1.00**	**1.00**	**–**	1.0%	0.3 to 3.1	**1.00**	**1.00**	–	25.3%	21.8 to 29.2	**1.00**	**1.00**	–	332, 412	133, 176	524, 706
5–9	1.2%	0.3 to 4.0	**3.21**	**1.78**	**0.20 to 15.5**	3.8%	1.3 to 10.8	**4.15**	**4.87**	**0.58 to 40.2**	39.6%	33.6 to 45.9	**1.93**	**1.50**	**1.01 to 2.21**	141, 200	84, 123	222, 314
10+	7.6%	4.8 to 11.7	**22.6**	**8.69**	**1.21 to 62.0**	12.3%	7.6 to 19.2	**14.6**	**19.80**	**3.03 to 129.**	49.2%	43.2 to 55.2	**2.86**	**2.23**	**1.45 to 3.42**	148, 224	121, 167	247, 342
Condom used for most recent sex with most recent partner																		
Yes	0.7%	0.2 to 2.0	**1.00**	1.00	–	4.3%	2.0 to 8.9	1.00	1.00	–	33.5%	29.7 to 37.6	1.00	1.00	–	301, 391	163, 221	491, 671
No	4.1%	2.6 to 6.4	**6.03**	3.59	0.77 to 16.6	7.4%	4.5 to 12.0	1.79	1.06	0.35 to 3.21	38.0%	33.9 to 42.4	1.22	0.97	0.70 to 1.34	283, 398	172, 237	458, 621
Concurrent partnerships in last year**																		
No	2.6%	1.6 to 4.2	1.00	1.00	–	6.7%	4.1 to 10.8	1.00	1.00	–	32.9%	29.6 to 36.3	**1.00**	1.00	–	418, 557	219, 298	676, 916
Yes	2.0%	0.7 to 5.4	0.75	0.18	0.04 to 0.71	6.5%	2.7 to 14.8	0.96	0.60	0.19 to 1.79	49.7%	41.0 to 58.4	**2.02**	1.52	0.92 to 2.50	82, 121	67, 92	136, 188
Unknown	1.7%	0.5 to 5.6	0.64	0.60	0.11 to 3.00	1.3%	0.3 to 5.5	0.19	0.06	0.00 to 0.71	38.5%	30.9 to 46.7	**1.28**	1.18	0.77 to 1.80	92, 122	52, 75	137, 193
Frequency of binge drinking																		
Never /≤monthly	1.1%	0.4 to 2.7	1.00	1.00	–	3.5%	1.6 to 7.7	1.00	1.00	–	28.1%	24.6 to 32.0	**1.00**	**1.00**	–	322, 421	146, 204	527, 715
Monthly	3.0%	1.4 to 6.2	2.79	1.47	0.33 to 6.48	4.0%	1.5 to 10.3	1.15	0.60	0.13 to 2.67	39.9%	33.4 to 46.7	**1.69**	**1.48**	**1.01 to 2.16**	125, 177	80, 113	202, 289
≥Weekly	3.8%	2.0 to 7.0	3.58	2.03	0.49 to 8.38	9.8%	5.6 to 16.6	2.98	1.23	0.48 to 3.14	43.2%	37.5 to 49.1	**1.94**	**1.50**	**1.04 to 2.15**	177, 241	117, 154	273, 370
Ever had any same sex experience/contact																		
No	2.3%	1.5 to 3.5	1.00	1.00	–	5.8%	3.8 to 8.9	1.00	1.00	–	33.9%	31.1 to 36.8	1.00	**1.00**	–	577, 762	311, 427	922, 1260
Yes	2.0%	0.4 to 9.0	0.90	0.31	0.02 to 4.16	5.1%	1.0 to 22.0	0.87	0.79	0.10 to 6.09	42.4%	32.2 to 53.4	1.44	**2.08**	**1.12 to 3.84**	47, 78	31, 44	80, 115

Variables in bold indicate those which are statistically significant (p<0.05).

95% CI of unadjusted OR and p values for unadjusted and adjusted OR are presented in full in the online supplementary material.

*N in column headings shows unweighted denominators. Total denominators by characteristic and in multivariable models vary due to item-missingness.

†AOR adjusted for all variables shown.

‡Results for recent diagnosis are not reported due to small sample size in Scotland and Wales when limited to those tested.

§IMD of LSOA of residence. IMD scores for England, Scotland and Wales were adjusted before being combined and assigned to quintiles, using the method described by Payne and Abel.^25^

¶Excludes 16 year olds.

**Among those with ≥1 sexual partner in last year.

††With two exceptions, all variables included in univariable models were included in multivariable models: number of sexual partners in the last year was not included due to collinearity with other sexual partnership variables; age left school was not included as data were unavailable for 16 year-olds.

AOR, adjusted OR; IMD, Index of Multiple Deprivation; LSOA, lower super output area.

**Figure 1:**
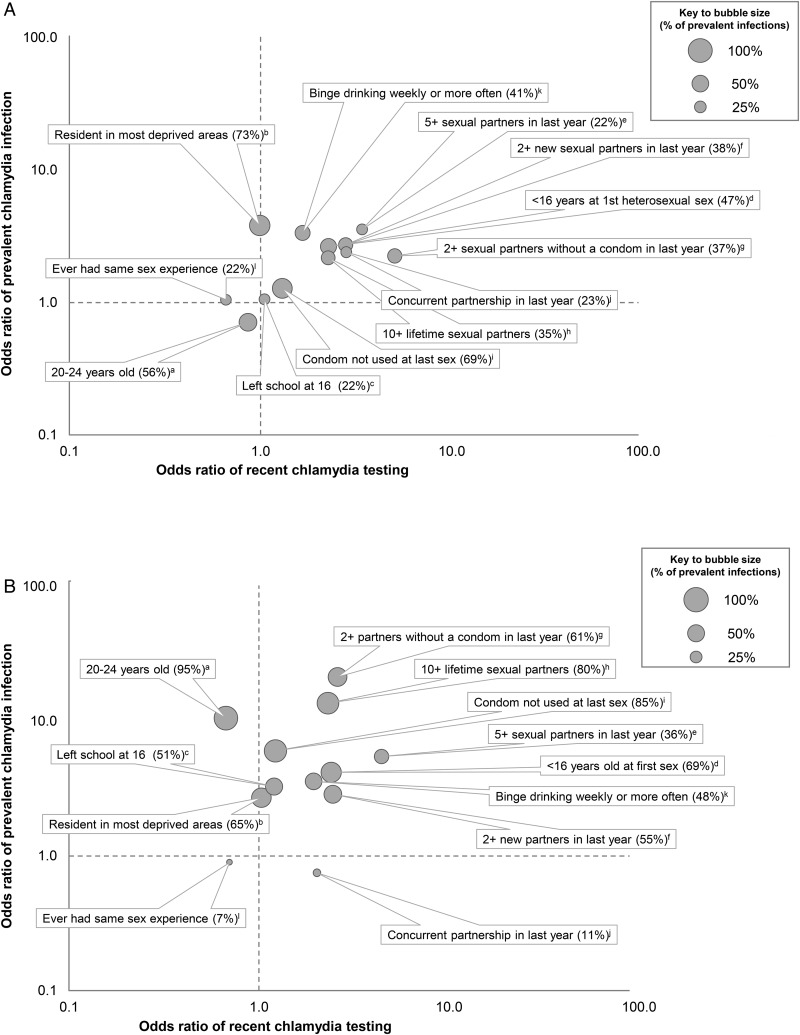
Bubble plot showing unadjusted ORs for prevalent chlamydia infection compared with recent testing by socio-demographic and behavioural factors, and proportion of prevalent infections in each group (16-year-old to 24-year-old sexually experienced women (A) and men (B)). Factors in the upper-right-hand quadrant are those where both the odds of prevalent infection and of testing were higher than the reference group. Factors in the upper-left-hand quadrant show those where the odds of prevalent infection were higher, but odds of testing were lower than the reference group (for ORs, 95% CIs and denominators, see tables 3 and 4). The area of the bubble and percentage in parentheses represents the proportion of individuals with a prevalent infection who reported the specified characteristic (for 95% CIs, see online supplementary table S1). Letters indicate reference groups: (a) 16–19 years old; (b) resident in lower super output area in the two least deprived quintiles, as measured by the Index of Multiple Deprivation; (c) left school at 17+ (among those aged ≥16); (d) 17+ years at first heterosexual sex; (e) 0 or 1 sexual partners in the last year; (f) 0 new sexual partners in the last year; (g) 0 sexual partners in the last year without a condom; (h) 1–9 lifetime sexual partners; (i) condom used at last sex; (j) no concurrent partnership in last year (among those with 1+ more sexual partners in last year); (k) reports binge drinking never or less than monthly; and (l) never had same sex contact/experience.

Figure 1 shows unadjusted ORs for prevalent infection and recent testing by socio-demographic and behavioural factors. Groups in the upper right hand quadrant are those where both the odds of prevalent infection and of testing were higher than the reference group. Groups in the upper-left-hand quadrant had higher odds of prevalent infection, but lower odds of testing than the reference group. Factors associated with recent testing were similar to those associated with prevalent infection, with some exceptions. Whereas women living in one of the two most deprived IMD quintiles had almost four times higher odds of prevalent infection versus those living in less deprived areas (OR 3.82, 95% CI 1.35 to 10.79), the odds of recent testing did not differ by deprivation (OR 0.99, 0.77 to 1.27). Among men, the odds of prevalent infection were higher among 20–24 vs 16–19 year olds (OR 10.6, 2.40 to 46.3), but odds of recent testing were lower in the older age group (OR 0.67, 0.44 to 0.84). In men, not having used a condom at last sex was associated with a sixfold increase in the odds of prevalent infection (OR 6.03, 1.87 to 19.42), but was not associated with recent testing (OR 1.22, 0.95 to 1.56). Similar patterns were seen when comparing adjusted ORs from multivariable models (tables 3 and 4).

Although the proportion recently tested was generally higher in those reporting risk factors for chlamydia, recent testing remained well below 100% in all socio-demographic and behavioural subgroups. For example, 30.0% of women and 53.7% of men with ≥2 new sexual partners in the last year and 25.8% of women and 51.2% of men reporting ≥2 sexual partners without a condom in the last year had not been recently tested (tables 3 and 4).

Among individuals with a prevalent chlamydia infection, 14% (95% CI 7% to 14%) had ever been diagnosed with chlamydia and 5% (2% to 17%) reported a diagnosis in the last year (indicating either repeat or persistent infections). Fifty per cent (35–64%) of those with a prevalent infection reported a recent chlamydia test (89% of whom did not report a recent diagnosis, thus indicating incident infections within the last year). Over two-thirds of prevalent infections were among individuals resident in one of the 40% most deprived LSOA. Infections in women were more evenly distributed by numbers of sexual partners than in men. For example, among men, 80% of those with a prevalent infection and 77% of those recently diagnosed reported ≥10 lifetime sexual partners versus only 25% of the population. In women, 35% of those with a prevalent infection reported ≥10 lifetime sexual partners versus 21% of the population (see online supplementary table S1).

## Discussion

### Principal findings

In 2010–2012, chlamydia was a common, and commonly diagnosed, infection among young adults in Britain. Diagnoses had arisen following both opportunistic screening and clinically indicated testing in roughly equal numbers. Living in more deprived areas was significantly associated with prevalent infection after adjusting for socio-demographic and behavioural factors. The proportion reporting chlamydia testing was generally greater among those reporting factors associated with chlamydia. However, substantial proportions of young adults reporting risk factors for chlamydia had not been recently tested.

### Strengths and limitations

The major strength of our study is that we used individual-level data from a nationally representative sample. We linked behavioural and biological data to examine a range of risk factors for different outcomes within the same survey and carried out multivariable analyses incorporating socio-demographic and behavioural data to minimise confounding of associations between predictor and outcome variables. There were some limitations. The number of participants aged under 25 limited statistical power to explore all associations of potential interest. For example, while recent testing was found to be higher in England than Scotland or Wales, consistent with chlamydia screening being offered opportunistically as part of an organised programme, the sample size was not large enough to determine whether factors associated with infection differed by country. Given the relatively small absolute numbers of prevalent infections, the proportions in specific subgroups should be interpreted with caution. Our findings may be affected by who agreed to take part in the survey or provide a urine sample. Survey weights were used to minimise bias, but unmeasured bias remains feasible. Our comparisons between risk factors for prevalent infection and recent testing may have been affected by the estimation of outcomes among different denominators. We explored this further in a sensitivity analysis, which showed no notable difference between ORs for testing when estimated in sexually experienced participants versus urine study participants (data not shown). A further limitation is the accuracy of self-reporting. Detailed questions were answered via self-completion, which we expect to have minimised social desirability bias.

Chlamydia infection was measured in urine. This may have missed some infections in women among whom vulvovaginal swabs demonstrate marginally higher sensitivity.^26^ Urine sampling will also have missed rectal infections, leading to underestimation of the total currently infected with chlamydia. However, the impact on our findings is likely minimal as men who have sex with men made up a small proportion of our sample.^27^

### Comparison to other studies/data

Estimates of chlamydia prevalence among young adults in Natsal-3 are comparable to those from other nationally representative surveys from Europe and high-income countries.^3^ Chlamydia prevalence is also similar to that reported in the previous Natsal (conducted in 1999–2001), where prevalence for 18–24 year olds was estimated at 3.0% in women and 2.7% in men.^22^ Comparisons between the surveys should be made with caution due to differences between them^17^ and because they were not powered to detect a change in prevalence. Recent testing was not associated with area-level deprivation in our study. This is contrary to an analysis of data from the southeast of England, which found higher rates of chlamydia screening in more deprived areas in 2008.^28^ This difference in findings may reflect the different study period, when screening coverage was lower, or regional variation in screening patterns.

National surveillance data on chlamydia tests and diagnoses among 15–24 year olds are available for England for the period covered by Natsal-3. The average coverage of chlamydia testing in England in 2010–2012 among 15–24 year olds was 40% in women and 20% in men.^14^ ^15^ This is lower than the 57% of women and 37% of men resident in England who reported a test in the last year in Natsal-3. Differences between denominators (all vs sexually experienced only) and age ranges (surveillance data for this period use partly aggregated data and are not available for 16–24 year olds) may partly explain these differences. Applying the proportion of 16–24 year olds with ≥1 sexual partner estimated in Natsal-3 (80%)^27^ to surveillance data results in an estimated coverage per year of 51% and 25% among sexually experienced women and men, respectively. This is more comparable but still somewhat lower than our estimates. This may indicate some residual bias arising from who took part in Natsal-3. Our findings on location of last test among those recently diagnosed are consistent with 2011 surveillance data, where 42% of diagnoses among 15–24 year olds were reported from GUM clinics, 15% from family planning services, 7% from GPs, 2% from education and 33% from other/unknown settings.^14^ The proportion of diagnoses from GPs was higher in Natsal-3 (27%) than in surveillance data. This may reflect the partially aggregate nature of surveillance data as a large proportion of diagnoses made in other/unknown settings are likely to be from GPs.

### Implications for chlamydia control in Britain

Encouragingly, those reporting risk factors for chlamydia were generally more likely to report having been recently tested. This is contrary to uptake patterns often seen in public health interventions, where those in most need are often least likely to access care.^29^ However, at least one-quarter of women and around half of men reporting a risk factor associated with prevalent infection had not been recently tested. This presents a clear potential for ongoing transmission of chlamydia from high risk but untested individuals. Almost all prevalent infections in men were among 20–24 year olds, less than a third of whom reported recent testing. As young women tend to have slightly older male partners,^30^ sexual mixing patterns by age may play a key role in transmission.

Our findings suggest that the likelihood of having an infection diagnosed and treated varies by deprivation, as although screening coverage was uniform by area-level deprivation, chlamydia prevalence was higher in those living in more deprived areas. This raises the question as to whether efforts to expand or intensify chlamydia screening should prioritise those living in more deprived areas to address this potential inequality. A high proportion of infections were found in those who had not used a condom at last sex, and around one-fifth of recent diagnoses were made following a test prompted by a partner having chlamydia, which emphasises the importance of condom use and partner notification in chlamydia prevention and control.

### Unanswered questions and future research

Increased screening and prevention efforts among individuals living in deprived areas and those reporting risk factors for chlamydia who are not regularly accessing screening may reduce the prevalence of undiagnosed infection and decrease transmission. The relative costs, feasibility and acceptability of different approaches to chlamydia screening warrant careful consideration in light of our findings.

**Key messages**

- Using a nationally representative sample of the British population, we compared factors associated with chlamydia prevalence, testing and diagnosis among 16-year-old to 24-year-old women and men.
- The proportion reporting chlamydia testing was generally greater among those reporting factors associated with chlamydia (eg, among those with more sexual partners).
- However, substantial proportions of young adults reporting risk factors for chlamydia had not been recently tested.
- Greater screening and prevention efforts among individuals living in deprived areas and those reporting risk factors for chlamydia who are not regularly accessing screening may reduce the prevalence of undiagnosed infection and decrease transmission.

## Supplementary Material

Web supplement
