# Supplementary material for: Is chlamydia screening and testing in Britain reaching young adults at risk of infection? Findings from the third National Survey of Sexual Attitudes and Lifestyles (Natsal-3)
Source: Sex Transm Infect. 2015 Aug 19;92(3):218–27. doi: 10.1136/sextrans-2015-052013 (PMC4853535; doi:10.1136/sextrans-2015-052013)
Supplement: Web supplement [file sextrans-2015-052013-s1.pdf]

Is chlamydia screening and testing in Britain reaching young adults at risk of infection? Findings from the third National Survey of Sexual Attitudes and Lifestyles (Natsal-3)

ONLINE SUPPLEMENTARY MATERIAL

Supplementary Figure S1: Flow chart showing participants included in analyses of self-reported testing, self-reported diagnosis and prevalent infection detected in urine among 16 to 24 year-old Natsal-3 participants

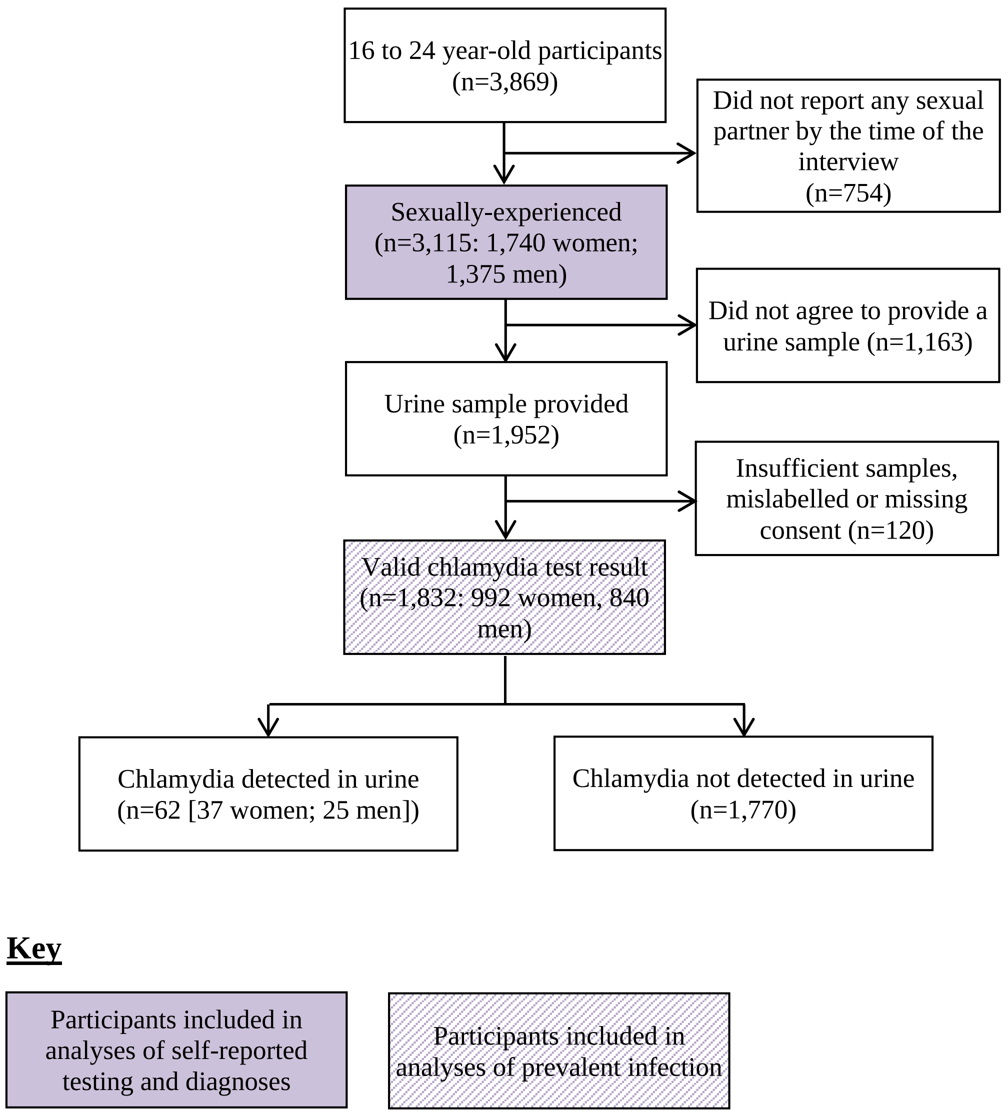

Supplementary Table S1: Distribution of selected demographic and behavioural characteristics among a) individuals with a prevalent chlamydia infection, b) individuals reporting a recent chlamydia diagnosis and c) the sexually-experienced population (16-24 year old sexually-experienced women).

	(a) Prevalent infection detected in urine (n=37)		(b) Diagnosed with chlamydia in the last year (n=54)		(c) Sexually-experienced population (n=1,740)	
	Percent	95% CI	Percent	95% CI	Percent	95% CI
**Age group**						
16-19	43.6%	(26.7-62.1)	40.5%	(26.6-56.2)	35.6%	(33.2-38.0)
20-24	56.4%	(37.9-73.3)	59.5%	(43.8-73.4)	64.4%	(62.0-66.8)
**Country**						
England	79.8%	(62.5-90.3)	92.1%	(80.8-97.0)	85.1%	(83.1-86.9)
Scotland	9.5%	(3.3-24.5)	3.4%	(0.7-14.3)	9.5%	(8.0-11.2)
Wales	10.8%	(3.8-27.0)	4.5%	(1.3-14.3)	5.4%	(4.4-6.7)
**IMD quintile of LSOA of residence[a]**						
2 least deprived quintiles	15.6%	(5.9-35.2)	30.9%	(18.4-46.9)	35.1%	(32.3-38.0)
middle quintile	11.0%	(4.5-24.7)	12.5%	(5.6-25.4)	19.5%	(17.3-21.9)
2 most deprived quintiles	73.4%	(54.9-86.3)	56.7%	(40.8-71.3)	45.4%	(42.5-48.4)
**Age left school[b]**						
17+	77.9%	(62.0-88.3)	74.9%	(60.2-85.4)	78.5%	(76.3-80.6)
16	22.1%	(11.7-38.0)	25.1%	(14.6-39.8)	21.5%	(19.4-23.7)
**Age at first heterosexual sex**						
17+	16.0%	(6.5-34.3)	19.5%	(8.9-37.7)	33.6%	(31.0-36.3)
16	37.3%	(21.0-57.1)	27.0%	(15.1-43.3)	29.5%	(27.0-32.0)
<16	46.8%	(28.8-65.6)	53.5%	(38.0-68.4)	36.9%	(34.3-39.6)
**Number of sexual partners in the last year**						
0 or 1	52.1%	(33.7-69.9)	31.10%	(18.2-47.9)	65.3%	(62.7-67.9)
2	19.4%	(8.6-38.0)	21.70%	(12.3-35.5)	15.0%	(13.3-17.0)
3 to 4	6.4%	(2.1-17.5)	23.60%	(13.6-37.7)	11.6%	(10.1-13.2)
5+	22.2%	(9.8-42.7)	23.60%	(12.2-40.6)	8.1%	(6.6-9.9)
**Number of new sexual partners in the last year**						
0	38.0%	(22.4-56.6)	21.9%	(12.0-36.7)	51.8%	(49.0-54.5)
1	24.4%	(10.9-46.0)	26.1%	(13.9-43.7)	27.6%	(25.2-30.1)
2+	37.6%	(21.7-56.6)	51.9%	(36.4-67.1)	20.6%	(18.4-23.1)
**Number of sexual partners in the last year without a condom**						
0	18.9%	(7.5-40.1)	11.4%	(3.9-28.6)	21.8%	(19.7-24.1)
1	44.1%	(26.9-62.9)	43.0%	(28.6-58.6)	60.6%	(58.0-63.2)
2+	36.9%	(20.6-57.0)	45.7%	(31.5-60.6)	17.5%	(15.6-19.6)
**Number of sexual partners over the lifetime**						
1 to 4	42.2%	(25.1-61.4)	16.1%	(8.3-28.8)	52.6%	(49.9-55.3)
5 to 9	22.6%	(11.3-40.1)	35.0%	(21.6-51.2)	26.3%	(24.0-28.8)
10+	35.2%	(19.2-55.3)	48.9%	(33.5-64.5)	21.1%	(19.0-23.4)
**Condom used for most recent sex with most recent partner**						
Yes	31.1%	(16.6-50.5)	21.9%	(10.7-39.7)	36.8%	(34.2-39.6)
No	68.9%	(49.5-83.4)	78.1%	(60.3-89.3)	63.2%	(60.4-65.8)
**Concurrent partnerships in last year [c]**						
No	65.0%	(45.4-80.6)	56.0%	(40.5-70.4)	76.2%	(73.9-78.3)
Yes	22.6%	(10.0-43.5)	22.8%	(11.8-39.5)	11.3%	(9.7-13.1)
Unknown	12.4%	(5.0-27.6)	21.2%	(11.7-35.4)	12.6%	(10.9-14.4)
**Frequency of binge drinking**						
never / less than monthly	50.5%	(32.4-68.6)	41.2%	(27.0-57.0)	62.3%	(59.7-64.9)
monthly	8.7%	(3.0-22.9)	26.4%	(14.7-43.0)	22.1%	(19.9-24.5)
weekly or more often	40.7%	(24.2-59.6)	32.4%	(18.8-49.8)	15.6%	(13.7-17.6)
**Ever had any same sex experience/contact**						
Yes	21.6%	(9.4-42.2)	28.1%	(16.5-43.6)	22.5%	(20.3-24.8)
No	78.4%	(57.8-90.6)	71.9%	(56.4-83.5)	77.5%	(75.2-79.7)
Bases (wt, unwt)		18, 37		28, 54		968, 1740

Supplementary Table S2: Distribution of selected demographic and behavioural characteristics among a) individuals with a prevalent chlamydia infection, b) individuals reporting a recent chlamydia diagnosis and c) the sexually-experienced population reporting selected demographic and behavioural characteristics (16-24 year old sexually-experienced men).

	**(a) Prevalent infection detected in urine (n=25)**		**(b) Diagnosed with chlamydia in the last year (n=27)**		**(c) Sexually-experienced population (n=1,375)**	
	Percent	95% CI	Percent	95% CI	Percent	95% CI
**Age group**						
16-19	5.5%	(1.2-22.5)	35.6%	(17.9-58.3)	37.3%	(34.4-40.2)
20-24	94.5%	(77.5-98.8)	64.4%	(41.7-82.1)	62.7%	(59.8-65.6)
**Country**						
England	71.8%	(47.3-87.9)	78.8%	(55.5-91.7)	85.7%	(82.7-88.2)
Scotland	24.2%	(9.5-49.3)	19.2%	(7.0-43.1)	8.8%	(6.8-11.4)
Wales	3.9%	(0.5-26.9)	2.0%	(0.2-14.5)	5.5%	(4.0-7.5)
**IMD quintile of LSOA of residence[a]**						
2 least deprived quintiles	22.0%	(7.3-50.1)	33.7%	(16.4-56.7)	36.9%	(33.6-40.2)
middle quintile	12.9%	(4.1-34.1)	15.3%	(5.1-38.0)	18.2%	(15.9-20.7)
2 most deprived quintiles	65.1%	(41.5-83.1)	51.0%	(29.9-71.8)	44.9%	(41.4-48.5)
**Age left school[b]**						
17+	49.3%	(27.7-71.1)	66.5%	(43.5-83.6)	75.4%	(72.6-77.9)
16	50.7%	(28.9-72.3)	33.5%	(16.4-56.5)	24.6%	(22.1-27.4)
**Age at first heterosexual sex**						
17+	15.1%	(4.9-37.9)	12.3%	(3.1-38.4)	35.1%	(32.1-38.2)
16	15.7%	(4.5-42.3)	20.6%	(7.7-44.7)	26.1%	(23.5-28.8)
<16	69.2%	(44.9-86.1)	67.1%	(43.1-84.6)	38.8%	(35.8-41.8)
**Number of sexual partners in the last year**						
0 or 1	37.4%	(18.2-61.6)	25.2%	(10.7-48.7)	57.30%	(54.3-60.3)
2	11.0%	(3.0-33.3)	3.5%	(0.7-15.0)	18.70%	(16.5-21.2)
3 to 4	15.6%	(5.1-38.5)	4.4%	(1.0-18.0)	13.60%	(11.7-15.7)
5+	36.1%	(17.6-59.8)	66.9%	(44.4-83.6)	10.40%	(8.8-12.2)
**Number of new sexual partners in the last year**						
0	34.1%	(16.0-58.5)	30.3%	(13.1-55.4)	42.1%	(39.0-45.2)
1	11.1%	(3.0-33.2)	23.3%	(9.8-45.9)	32.6%	(29.7-35.6)
2+	54.8%	(32.0-75.7)	46.5%	(26.3-67.9)	25.3%	(22.8-28.0)
**Number of sexual partners in the last year without a condom**						
0	4.8%	(1.0-19.9)	8.2%	(1.6-33.0)	33.1%	(30.2-36.1)
1	34.7%	(16.1-59.5)	39.7%	(21.1-61.8)	47.5%	(44.4-50.5)
2+	60.5%	(36.7-80.2)	52.1%	(30.7-72.8)	19.4%	(17.2-21.8)
**Number of sexual partners over the lifetime**						
1 to 4	8.50%	(1.8-32.5)	6.5%	(1.8-21.3)	52.70%	(49.8-55.7)
5 to 9	11.60%	(3.0-35.8)	16.7%	(5.5-41.1)	22.40%	(20.0-25.0)
10+	79.90%	(55.8-92.6)	76.7%	(53.8-90.3)	24.90%	(22.5-27.4)
**Condom used for most recent sex with most recent partner**						
Yes	15.5%	(4.9-39.3)	35.3%	(17.2-58.9)	51.7%	(48.6-54.8)
No	84.5%	(60.7-95.1)	64.7%	(41.1-82.8)	48.3%	(45.2-51.4)
**Concurrent partnerships in last year[c]**						
No	77.6%	(55.8-90.5)	74.5%	(52.3-88.7)	71.2%	(68.4-74.0)
Yes	11.4%	(3.7-30.3)	22.0%	(9.0-44.6)	14.3%	(12.3-16.6)
Unknown	11.0%	(3.0-33.3)	3.5%	(0.7-15.0)	14.5%	(12.5-16.7)
**Frequency of binge drinking**						
never / less than monthly	25.1%	(9.9-50.6)	25.9%	(11.2-49.1)	52.6%	(49.6-55.5)
monthly	26.7%	(12.0-49.5)	16.2%	(5.8-37.7)	20.2%	(17.9-22.6)
weekly or more often	48.2%	(26.6-70.4)	57.9%	(35.8-77.3)	27.3%	(24.7-30.0)
**Ever had any same sex experience/contact**						
Yes	6.8%	(1.3-29.3)	8.2%	(1.6-33.0)	8.0%	(6.5-9.8)
No	93.2%	(70.7-98.7)	91.8%	(67.0-98.4)	92.0%	(90.2-93.5)
Bases (wt, unwt)		14, 25		20, 27		1003, 1375

N in column headings shows unweighted denominators. Total denominators by characteristic vary due to item-missingness.
[a]IMD: Index of multiple deprivation of LSOA (lower super output area) of residence. IMD scores for England, Scotland and Wales were adjusted before being combined and assigned to quintiles, using the method described by Payne and Abel[39]; [b]Excludes 16 year olds;[c]Among those with 1+ more sexual partners in last year.

Supplementary Table S3: Percentage, unadjusted and adjusted odds ratios for prevalent chlamydia infection, self-reported diagnosis in the last year and self-reported testing by sociodemographic and behavioural factors (sexually-experienced 16-24 year-old women)

	Prevalent infection detected in urine (n=992)								Diagnosed with chlamydia in the last year (among those tested in the last year) (n=940)								Tested for chlamydia in the last year (n=1,736)								Denominator (weighted, unweighted) [a]		
	%	(95% CI)	OR	(95% CI)	p	AOR[b]	(95% CI)	p	%	(95% CI)	OR	(95% CI)	p	AOR[b]	(95% CI)	p	%	(95% CI)	OR	(95% CI)	p	AOR[b]	(95% CI)	p	Infection	Diagnosis	Tested
**Age group**																											
16-19	3.8%	(2.2-6.3)	1.00	-	0.36	1.00	-	0.50	6.0%	(3.8-9.2)	1.00	-	0.62	1.00-		0.58	56.6%	(52.5-60.6)	1.00	-	0.16	1.00	-	0.15	214, 395	193, 375	343, 672
20-24	2.7%	(1.7-4.3)	0.71	(0.35-1.46)		0.71	(0.27-1.87)		5.1%	(3.4-7.6)	0.86	(0.46-1.60)		0.80	(0.35-1.78)		52.8%	(49.2-56.4)	0.86	(0.69-1.06)		0.82	(0.62-1.07)		383, 597	329, 565	623, 1064
**Country[c]**																											
England	2.9%	(2.0-4.3)	1.00	-	0.53	1.00	-	0.48									57.1%	(54.1-60.1)	1.00	-	**<0.01**	1.00	-	**<0.01**	504, 817	469, 832	823, 1452
Scotland	3.1%	(1.1-8.6)	1.08	(0.35-3.33)		1.34	(0.43-4.14)										32.4%	(24.4-41.5)	0.36	(0.24-0.54)		0.29	(0.18-0.45)		56, 103	30, 58	91, 178
Wales	5.3%	(1.9-13.8)	1.87	(0.63-5.60)		1.88	(0.63-5.54)										45.6%	(36.2-55.4)	0.63	(0.42-0.94)		0.53	(0.32-0.85)		37, 72	24, 50	52, 106
**IMD quintile of LSOA of residence[d]**																											
2 least deprived	1.3%	(0.5-3.4)	1.00	-	**0.01**	1.00	-	**0.01**	4.8%	(2.8-8.1)	1.00	-	0.23	1.00-		0.36	54.2%	(49.5-58.8)	1.00	-	0.99	1.00	-	0.94	213, 355	183, 319	338, 595
Middle quintile	1.8%	(0.8-4.2)	1.37	(0.38-4.90)		1.40	(0.39-4.98)		3.5%	(1.6-7.3)	0.71	(0.27-1.86)		1.06	(0.37-3.04)		54.4%	(48.0-60.7)	1.01	(0.74-1.38)		1.03	(0.71-1.48)		111, 174	102, 176	189, 324
2 most deprived	4.9%	(3.3-7.3)	3.82	(1.35-10.8)		4.23	(1.53-11.6)		6.8%	(4.6-10.0)	1.46	(0.73-2.93)		1.70	(0.73-3.91)		54.0%	(49.8-58.2)	0.99	(0.77-1.27)		0.97	(0.73-1.29)		273, 463	236, 445	439, 817
**Age left school[e]**																											
17+	3.2%	(2.1-4.8)	1.00	-	0.88				5.2%	(3.6-7.5)	1.00	-	0.58				54.3%	(51.0-57.6)	1.00	-	0.70				445, 700	387, 658	715, 1217
16	3.4%	(1.9-6.0)	1.06	(0.50-2.22)					6.2%	(3.7-10.5)	1.21	(0.62-2.37)					55.5%	(50.1-60.8)	1.05	(0.82-1.35)					120, 229	109, 228	196, 405
**Age at first heterosexual sex**																											
17+	1.6%	(0.7-3.7)	1.00	-	0.15	1.00	-	0.42	4.1%	(1.8-8.9)	1.00	-	0.37	1.00-		0.93	43.9%	(38.7-49.1)	1.00	-	**<0.01**	1.00	-	0.05	188, 246	137, 215	313, 489
16	3.9%	(2.1-6.9)	2.52	(0.86-7.36)		2.20	(0.67-7.17)		5.0%	(2.8-8.9)	1.24	(0.44-3.55)		0.80	(0.26-2.42)		56.4%	(51.6-61.1)	1.66	(1.25-2.20)		1.39	(0.99-1.92)		178, 304	154, 272	273, 503
<16	4.0%	(2.4-6.6)	2.65	(0.95-7.36)		1.82	(0.60-5.42)		6.9%	(4.8-9.9)	1.76	(0.70-4.41)		0.89	(0.35-2.25)		63.9%	(59.8-67.9)	2.27	(1.75-2.94)		1.44	(1.05-1.97)		213, 415	220, 429	344, 680
**Number of sexual partners in the last year**																											
0 or 1	2.5%	(1.5-4.0)	1.00	-	**0.03**				3.0%	(1.7-5.4)	1.00	-	**0.01**				46.6%	(43.3-50.0)	1.00	-	**<0.01**				387, 600	291, 507	624, 1096
2	3.9%	(1.8-8.5)	1.62	(0.63-4.15)					6.6%	(3.7-11.6)	2.25	(0.98-5.18)					65.2%	(58.8-71.1)	2.15	(1.59-2.89)					90, 161	93, 178	143, 275
3 to 4	1.9%	(0.7-5.1)	0.75	(0.24-2.33)					8.8%	(5.2-14.6)	3.08	(1.34-7.05)					69.7%	(62.0-76.4)	2.63	(1.82-3.80)					63, 127	76, 146	111, 210
5+	8.3%	(3.9-16.8)	3.57	(1.39-9.17)					11.6%	(6.2-20.8)	4.19	(1.69-10.4)					74.8%	(64.4-83.0)	3.40	(2.05-5.65)					49, 93	58, 101	77, 135
**Number of new sexual partners in the last year**																											
0	2.2%	(1.3-3.7)	1.00	-	**0.03**	1.00	-	0.70	2.8%	(1.5-5.0)	1.00	-	**<0.01**	1.00-		0.11	45.6%	(41.9-49.3)	1.00	-	**<0.01**	1.00	-	**<0.01**	313, 495	226, 397	495, 873
1	2.8%	(1.2-6.3)	1.26	(0.47-3.41)		1.17	(0.38-3.52)		4.8%	(2.5-9.1)	1.76	(0.71-4.38)		1.89	(0.69-5.16)		59.2%	(54.0-64.2)	1.73	(1.34-2.23)		1.69	(1.25-2.27)		160, 263	156, 287	264, 485
2+	5.9%	(3.5-9.8)	2.73	(1.26-5.93)		1.65	(0.50-5.39)		10.7%	(7.3-15.6)	4.23	(2.04-8.79)		3.09	(1.07-8.86)		70.0%	(63.8-75.6)	2.79	(2.03-3.84)		1.46	(0.95-2.21)		118, 225	137, 249	197, 359

**Number of sexual partners in the last year without a condom**																		
0	2.9% (1.2-7.1)	1.00 -	**0.03**	1.00 -	0.18	4.2%(1.5-11.6)	1.00 -	**<0.01**	1.00-	0.84	36.4%(30.8-42.3)	1.00 -	**<0.01**		0.05	120, 173	76, 130	210, 361
1	2.2% (1.4-3.6)	0.76 (0.26-2.17)		0.34 (0.10-1.10)		3.8%(2.4-6.1)	0.90(0.27-2.94)		0.72(0.15-3.21)		54.7%(51.4-58.0)	2.11(1.61-2.78)		1.52(1.03-2.24)		368, 606	319, 567	585, 1049
2+	6.3% (3.5-11.2)	2.25 (0.73-6.93)		0.49 (0.12-1.83)		10.3%(7.0-15.0)	2.60(0.80-8.46)		0.90(0.18-4.49)		74.2%(68.3-79.4)	5.04(3.47-7.32)		1.86(1.09-3.15)		108, 212	126, 241	169, 322
**Number of lifetime sexual partners**																		
1 to 4	2.4% (1.4-4.2)	1.00 -	0.14	1.00 -	0.67	2.1%(1.1-4.0)	1.00 -	**<0.01**	1.00-	0.08	43.5%(40.0-47.1)	1.00 -	**<0.01**	1.00 -	**<0.01**	321, 482	218, 391	503, 894
5 to 9	2.8% (1.4-5.2)	1.15 (0.48-2.75)		0.87 (0.32-2.32)		6.2%(3.7-10.3)	3.07(1.33-7.07)		2.40(0.90-6.37)		63.8%(58.5-68.7)	2.29(1.76-2.96)		1.96(1.43-2.69)		150, 267	161, 289	252, 453
10+	5.4% (2.9-9.7)	2.29 (0.97-5.39)		1.39 (0.45-4.18)		9.9%(6.5-14.7)	5.12(2.31-11.3)		3.76(1.19-11.8)		69.6%(63.9-74.7)	2.97(2.22-3.98)		2.11(1.41-3.13)		121, 234	141, 254	202, 373
**Condom used for most recent sex with most recent partner**																		
Yes	2.8% (1.5-5.3)	1.00 -	0.53	1.00 -	0.29	3.4%(1.6-7.0)	1.00 -	0.14	1.00-	0.14	51.6%(46.9-56.3)	1.00 -	**0.03**	1.00 -	0.97	202, 314	170, 303	330, 586
No	3.6% (2.4-5.4)	1.28 (0.59-2.80)		1.59 (0.67-3.74)		6.3%(4.4-8.9)	1.90(0.81-4.49)		1.88(0.81-4.32)		58.0%(54.6-61.3)	1.30(1.03-1.63)		1.01(0.74-1.35)		352, 613	328, 592	567, 1027
**Concurrent partnerships in last year**[f]																		
No	2.7% (1.8-4.2)	1.00 -	0.19	1.00 -	0.85	4.3%(2.9-6.4)	1.00 -	0.13	1.00-	0.46	51.0%(47.8-54.2)	1.00 -	**<0.01**	1.00 -	0.05	439, 706	361, 639	710, 1256
Yes	6.3% (2.9-13.4)	2.40 (0.94-6.15)		1.34 (0.48-3.70)		8.1%(4.1-15.3)	1.95(0.84-4.54)		0.75(0.27-2.00)		74.6%(67.1-80.8)	2.81(1.93-4.09)		1.46(0.93-2.28)		66, 134	78, 146	105, 196
Unknown	3.1% (1.3-7.2)	1.14 (0.43-3.04)		1.13 (0.38-3.31)		7.8%(4.3-13.9)	1.88(0.90-3.94)		1.46(0.62-3.38)		64.6%(57.5-71.2)	1.76(1.27-2.42)		1.53(1.02-2.28)		73, 127	76, 144	117, 226
**Frequency of binge drinking**																		
never / <u><</u>monthly	2.5% (1.5-4.1)	1.00 -	**<0.01**	1.00 -	**0.01**	3.7%(2.4-5.7)	1.00 -	**0.03**	1.00-	0.28	52.1%(48.7-55.5)	1.00 -	**0.01**	1.00 -	0.08	373, 598	312, 561	601, 1085
monthly	1.2% (0.5-3.3)	0.49 (0.16-1.52)		0.46 (0.13-1.51)		6.6%(3.6-11.8)	1.85(0.85-4.01)		1.91(0.74-4.89)		52.4%(46.6-58.2)	1.01(0.78-1.32)		0.75(0.54-1.02)		130, 226	112, 200	214, 375
<u>></u>weekly	7.9% (4.7-13.1)	3.35 (1.55-7.25)		2.51 (1.08-5.76)		9.4%(5.2-16.3)	2.69(1.25-5.80)		2.06(0.75-5.61)		64.4%(57.6-70.7)	1.66(1.22-2.27)		1.16(0.81-1.64)		95, 168	97, 177	151, 274
**Ever had any same sex experience/ contact**																		
No	3.1% (2.1-4.4)	1.00 -	0.92	1.00 -	0.49	5.3%(3.7-7.5)	1.00 -	0.72	1.00-	0.44	51.9%(48.8-55.0)	1.00 -	**<0.01**	1.00 -	0.24	473, 750	388, 704	749, 1352
Yes	3.2% (1.4-7.1)	1.05 (0.42-2.63)		0.74 (0.30-1.76)		5.9%(3.5-10.0)	1.13(0.57-2.24)		0.71(0.30-1.68)		61.9%(56.0-67.5)	1.51(1.15-1.98)		1.21(0.88-1.65)		124, 242	134, 236	218, 384

[a]N in column headings shows unweighted denominators. Total denominators by characteristic and in multivariable models vary due to item-missingness..[b]AOR: Adjusted odds ratios, adjusted for all variables shown. [c]Results for recent diagnosis are not reported due to small sample size in Scotland and Wales when limited to those tested. [d]IMD: Index of multiple deprivation of LSOA (lower super output area) of residence. IMD scores for England, Scotland and Wales were adjusted before being combined and assigned to quintiles, using the method described by Payne and Abel[24]. [e]Excludes 16 year-olds. [f]Among those with <u>></u>1 sexual partner in last year.

Supplementary Table S4: Percentage, unadjusted and adjusted odds ratios for prevalent chlamydia infection, self-reported diagnosis in the last year and self-reported testing by sociodemographic and behavioural factors (sexually-experienced 16-24 year-old men)

	Prevalent infection detected in urine (n=840)								Diagnosed with chlamydia in the last year (among those tested in the last year) (n=471)								Tested for chlamydia in the last year (n=1,375)								Denominator (weighted, unweighted) [a]		
	%	(95% CI)	OR	(95% CI)	p	AOR[b]	(95% CI)	p	%	(95% CI)	OR	(95% CI)	p	AOR[b]	(95% CI)	p	%	(95% CI)	OR	(95% CI)	p	AOR[b]	(95% CI)	p	Infection	Diagnosis	Tested
**Age group**																											
16-19	0.3%	(0.1-1.4)	1.00	-	**<0.01**	1.00	-	**0.02**	4.7%	(2.4-9.0)	1.00	-	0.41	1.00	-	0.61	40.4%	(35.9-45.1)	1.00	-	**<0.01**	1.00	-	**<0.01**	234, 343	151, 226	374, 582
20-24	3.4%	(2.2-5.2)	10.6	(2.40-46.3)		7.54	(1.37-41.3)		6.7%	(3.9-11.1)	1.46	(0.59-3.58)		0.76	(0.26-2.15)		31.1%	(27.8-34.7)	0.67	(0.52-0.86)		0.53	(0.37-0.73)		391, 497	192, 245	629, 793
**Country[c]**																											
England	1.9%	(1.2-3.0)	1.00	-	0.12	1.00	-	0.27									37.3%	(34.3-40.3)	1.00	-	**<0.01**	1.00	-	**<0.01**	532, 719	316, 440	859, 1181
Scotland	5.7%	(2.1-14.3)	3.13	(1.04-9.45)		3.16	(0.78-12.8)										22.2%	(14.0-33.5)	0.48	(0.27-0.85)		0.33	(0.16-0.64)		60, 72	20, 22	89, 111
Wales	1.7%	(0.2-12.1)	0.88	(0.11-7.02)		1.20	(0.18-7.63)										12.8%	(6.9-22.3)	0.25	(0.13-0.48)		0.19	(0.08-0.40)		33, 49	7, 9	55, 83
**IMD quintile of LSOA of residence[d]**																											
2 least deprived	1.3%	(0.4-3.6)	1.00	-	0.14	1.00	-	**0.04**	5.2%	(2.5-10.5)	1.00	-	0.85	1.00	-	0.85	34.5%	(30.0-39.2)	1.00	-	0.89	1.00	-	0.75	241, 315	127, 180	369, 509
Middle quintile	1.6%	(0.6-4.4)	1.24	(0.26-5.88)		1.01	(0.15-6.68)		5.0%	(1.7-13.8)	0.96	(0.26-3.58)		0.68	(0.15-2.97)		33.3%	(27.4-39.9)	0.95	(0.67-1.35)		1.04	(0.70-1.52)		114, 164	60, 86	183, 263
2 most deprived	3.4%	(2.1-5.6)	2.71	(0.83-8.82)		3.75	(1.11-12.5)		6.5%	(3.6-11.4)	1.26	(0.48-3.33)		1.06	(0.42-2.64)		35.2%	(31.1-39.5)	1.03	(0.78-1.36)		1.13	(0.82-1.53)		269, 361	155, 205	450, 603
**Age left school[e]**																											
17+	1.6%	(0.9-2.7)	1.00	-	**0.01**				5.3%	(3.1-9.0)	1.00	-	0.49				33.6%	(30.4-37.1)	1.00	-	0.21				439, 568	233, 304	703, 927
16	5.0%	(2.7-9.2)	3.28	(1.38-7.82)					7.2%	(3.6-13.8)	1.38	(0.55-3.45)					37.8%	(32.3-43.5)	1.20	(0.91-1.58)					143, 206	87, 134	230, 334
**Age at first heterosexual sex**																											
17+	1.0%	(0.3-2.8)	1.00	-	**0.03**	1.00	-	0.67	2.8%	(0.7-9.9)	1.00	-	0.26	1.00	-	0.74	25.6%	(21.4-30.3)	1.00	-	**<0.01**	1.00	-	0.05	210, 245	87, 112	340, 431
16	1.5%	(0.5-4.6)	1.49	(0.31-7.15)		1.14	(0.27-4.74)		4.7%	(1.8-11.8)	1.75	(0.33-9.27)		1.14	(0.24-5.30)		33.4%	(27.9-39.4)	1.46	(1.03-2.06)		1.13	(0.75-1.67)		148, 205	84, 108	253, 351
<16	4.0%	(2.4-6.5)	4.18	(1.31-13.3)		1.65	(0.55-4.90)		7.8%	(4.7-12.6)	2.99	(0.70-12.7)		1.58	(0.37-6.62)		45.3%	(40.7-49.9)	2.40	(1.78-3.23)		1.53	(1.07-2.19)		238, 352	167, 243	376, 539
**Number of sexual partners in the last year**																											
0 or 1	1.5%	(0.7-3.0)	1.00	-	**0.01**				3.4%	(1.5-7.8)	1.00	-	**<0.01**				26.0%	(22.6-29.7)	1.00	-	**<0.01**				359, 466	145, 196	568, 768
2	1.3%	(0.4-4.2)	0.86	(0.21-3.56)					0.9%	(0.2-3.9)	0.27	(0.05-1.41)					40.3%	(33.2-47.7)	1.92	(1.34-2.75)					123, 159	74, 99	185, 251
3 to 4	3.1%	(1.1-8.5)	2.16	(0.60-7.78)					1.5%	(0.4-6.2)	0.44	(0.08-2.30)					43.0%	(35.5-50.9)	2.15	(1.49-3.10)					70, 110	57, 83	134, 194
5+	7.5%	(3.7-14.6)	5.47	(1.89-15.9)					21.2%	(12.9-32.7)	7.54	(2.66-21.3)					60.9%	(51.8-69.3)	4.42	(2.93-6.67)					67, 100	63, 89	103, 146
**Number of new sexual partners in the last year**																											
0	1.8%	(0.9-3.8)	1.00	-	**0.01**	1.00	-	0.49	5.5%	(2.3-12.6)	1.00	-	0.37	1.00	-	0.31	26.0%	(22.0-30.5)	1.00	-	**<0.01**	1.00	-	0.37	263, 335	108, 136	416, 540
1	0.8%	(0.2-2.5)	0.42	(0.10-1.72)		0.33	(0.05-2.06)		4.0%	(1.7-9.0)	0.71	(0.21-2.47)		1.13	(0.09-13.9)		36.7%	(31.8-41.8)	1.64	(1.21-2.24)		1.28	(0.88-1.85)		203, 270	115, 161	323, 452
2+	5.1%	(2.9-8.8)	2.87	(1.08-7.63)		0.47	(0.09-2.45)		8.0%	(4.5-13.7)	1.48	(0.50-4.35)		2.87	(0.26-30.7)		46.3%	(40.7-52.0)	2.45	(1.78-3.37)		1.06	(0.67-1.68)		152, 229	115, 170	251, 366

**Number of sexual partners in the last year without a condom**																		
0	0.3% (0.1-1.3)	1.00 -	**<0.01**	1.00 -	0.09	1.8% (0.4-8.4)	1.00 -	**0.04**	1.00 -	0.45	27.0% (22.4-32.1)	1.00 -	**<0.01**	1.00 -	0.49	205, 248	88, 115	331, 450
1	1.7% (0.8-3.6)	5.26 (1.05-26.2)		1.23 (0.09-15.2)		4.9% (2.6-8.9)	2.75 (0.50-15.0)		0.78 (0.19-3.15)		34.3% (30.2-38.7)	1.41 (1.02-1.96)		1.12 (0.72-1.71)		287, 396	160, 222	475, 640
2+	6.5% (3.9-10.9)	21.3 (4.67-97.2)		4.95 (0.42-57.9)		10.9% (6.0-19.0)	6.51 (1.19-35.6)		0.46 (0.11-1.83)		48.8% (42.3-55.4)	2.59 (1.81-3.70)		1.37 (0.80-2.34)		130, 194	95, 134	194, 281
**Number of lifetime sexual partners**																		
1 to 4	0.4% (0.1-1.5)	1.00 -	**<0.01**	1.00 -	**0.03**	1.0% (0.3-3.1)	1.00 -	**<0.01**	1.00 -	**<0.01**	25.3% (21.8-29.2)	1.00 -	**<0.01**	1.00 -	**<0.01**	332, 412	133, 176	524, 706
5 to 9	1.2% (0.3-4.0)	3.21 (0.47-21.9)		1.78 (0.20-15.5)		3.8% (1.3-10.8)	4.15 (0.82-21.0)		4.87 (0.58-40.2)		39.6% (33.6-45.9)	1.93 (1.39-2.69)		1.50 (1.01-2.21)		141, 200	84, 123	222, 314
10+	7.6% (4.8-11.7)	22.6 (4.92-104)		8.69 (1.21-62.0)		12.3% (7.6-19.2)	14.6 (3.89-54.5)		19.80 (3.03-129.)		49.2% (43.2-55.2)	2.86 (2.09-3.92)		2.23 (1.45-3.42)		148, 224	121, 167	247, 342
**Condom used for most recent sex with most recent partner**																		
Yes	0.7% (0.2-2.0)	1.00 -	**<0.01**	1.00 -	0.10	4.3% (2.0-8.9)	1.00 -	0.22	1.00 -	0.91	33.5% (29.7-37.6)	1.00 -	0.13	1.00 -	0.87	301, 391	163, 221	491, 671
No	4.1% (2.6-6.4)	6.03 (1.87-19.4)		3.59 (0.77-16.6)		7.4% (4.5-12.0)	1.79 (0.70-4.59)		1.06 (0.35-3.21)		38.0% (33.9-42.4)	1.22 (0.95-1.56)		0.97 (0.70-1.34)		283, 398	172, 237	458, 621
**Concurrent partnerships in last year[f]**																		
No	2.6% (1.6-4.2)	1.00 -	0.74	1.00 -	0.05	6.7% (4.1-10.8)	1.00 -	0.10	1.00 -	0.07	32.9% (29.6-36.3)	1.00 -	**<0.01**	1.00 -	0.24	418, 557	219, 298	676, 916
Yes	2.0% (0.7-5.4)	0.75 (0.24-2.37)		0.18 (0.04-0.71)		6.5% (2.7-14.8)	0.96 (0.34-2.72)		0.60 (0.19-1.79)		49.7% (41.0-58.4)	2.02 (1.37-2.96)		1.52 (0.92-2.50)		82, 121	67, 92	136, 188
Unknown	1.7% (0.5-5.6)	0.64 (0.17-2.37)		0.60 (0.11-3.00)		1.3% (0.3-5.5)	0.19 (0.04-0.88)		0.06 (0.00-0.71)		38.5% (30.9-46.7)	1.28 (0.88-1.86)		1.18 (0.77-1.80)		92, 122	52, 75	137, 193
**Frequency of binge drinking**																		
never / <u><</u>monthly	1.1% (0.4-2.7)	1.00 -	0.08	1.00 -	0.62	3.5% (1.6-7.7)	1.00 -	0.07	1.00 -	0.46	28.1% (24.6-32.0)	1.00 -	**<0.01**	1.00 -	**0.04**	322, 421	146, 204	527, 715
monthly	3.0% (1.4-6.2)	2.79 (0.85-9.18)		1.47 (0.33-6.48)		4.0% (1.5-10.3)	1.15 (0.31-4.18)		0.60 (0.13-2.67)		39.9% (33.4-46.7)	1.69 (1.21-2.37)		1.48 (1.01-2.16)		125, 177	80, 113	202, 289
≥weekly	3.8% (2.0-7.0)	3.58 (1.17-11.0)		2.03 (0.49-8.38)		9.8% (5.6-16.6)	2.98 (1.07-8.34)		1.23 (0.48-3.14)		43.2% (37.5-49.1)	1.94 (1.44-2.62)		1.50 (1.04-2.15)		177, 241	117, 154	273, 370
**Ever had any same sex experience/ contact**																		
No	2.3% (1.5-3.5)	1.00 -	0.89	1.00 -	0.37	5.8% (3.8-8.9)	1.00 -	0.87	1.00 -	0.82	33.9% (31.1-36.8)	1.00 -	0.11	1.00 -	**0.02**	577, 762	311, 427	922, 1260
Yes	2.0% (0.4-9.0)	0.90 (0.18-4.48)		0.31 (0.02-4.16)		5.1% (1.0-22.0)	0.87 (0.17-4.54)		0.79 (0.10-6.09)		42.4% (32.2-53.4)	1.44 (0.92-2.26)		2.08 (1.12-3.84)		47, 78	31, 44	80, 115

[a]N in column headings shows unweighted denominators. Total denominators by characteristic and in multivariable models vary due to item-missingness..[b]AOR: Adjusted odds ratios, adjusted for all variables shown. [c]Results for recent diagnosis are not reported due to small sample size in Scotland and Wales when limited to those tested. [d]IMD: Index of multiple deprivation of LSOA (lower super output area) of residence. IMD scores for England, Scotland and Wales were adjusted before being combined and assigned to quintiles, using the method described by Payne and Abel[24]. [e]Excludes 16 year-olds. [f]Among those with ≥1 sexual partner in last year.
